# Estimating a minimum clinically important difference for the Developmental Behaviour Checklist – parent report

**DOI:** 10.3389/fpsyt.2025.1612911

**Published:** 2025-08-15

**Authors:** Daniel L. Sutherland, Emma L. Taylor, Kylie M. Gray, Richard P. Hastings, Amanda Allard, Joanna Carr, Joanna Griffin, Nicola McMeekin, Elizabeth Randell, Daisy Russell, Bronwen Willoughby-Richards, Jeanne Wolstencroft, Paul A. Thompson

**Affiliations:** ^1^ Centre for Research in Intellectual and Developmental Disabilities (CIDD), University of Warwick, Coventry, United Kingdom; ^2^ Intellectual Disabilities Research Institute (IDRIS), School of Social Policy and Society, University of Birmingham, Birmingham, United Kingdom; ^3^ Department of Psychiatry, School of Clinical Sciences at Monash Health, Monash University, Melbourne, VIC, Australia; ^4^ Council for Disabled Children, London, United Kingdom; ^5^ School of Health & Wellbeing, University of Glasgow, Glasgow, United Kingdom; ^6^ Centre for Trials Research, Cardiff University, Cardiff, United Kingdom; ^7^ The Great Ormond Street Institute of Child Health, University College London, London, United Kingdom

**Keywords:** intellectual disabilities, autism, minimum clinically important difference (MCID), meta analysis, Developmental Behavior Checklist (DBC)

## Abstract

**Background:**

Evaluating the effectiveness of interventions relies on understanding what change in a main outcome is sufficient to be considered meaningful. Our aim was to estimate a Minimum Clinically Important Difference (MCID) for the Developmental Behaviour Checklist, parent-report (DBC-P)- a measure of behavioural and emotional problems in children and adolescents with intellectual disabilities.

**Methods:**

We generated distribution-based estimates through meta-analysis of intervention evaluations using the DBC-P as an outcome measure. We also generated anchor-based estimates using case scenarios with 10 parent carers and 21 professionals working with people with intellectual disabilities.

**Results:**

21 studies were included in the meta-analyses and indicated an average DBC total raw score decrease of 3.01 or 4.73 (depending on analytic methods) in randomised controlled trials, and an average decrease of 9.16 points in pre-post designs. Parent carers provided a median MCID estimate of 6 (IQR 4, 7) and professionals provided a median estimate of 8 (IQR 5, 14).

**Conclusions:**

These findings contextualise DBC-P score changes in relation to outcomes from other interventions and parent carer and professional views. Which MCID value to choose depends on what factors are prioritised for an intervention.

## Introduction

Children with intellectual disabilities are more likely to display elevated behavioural and emotional problems compared with children without intellectual disabilities (1, 2). Moreover, these behavioural and emotional difficulties are often persistent over time (3–5) and, along with the intrinsic importance of directly reducing the distress of the child with an intellectual disability, are associated with a range of other outcomes such as out-of-home care (6), psychotropic polypharmacy (7, 8), and poorer psychological outcomes for family members (3, 9). Reducing behavioural and emotional problems amongst children with intellectual disabilities is, therefore, a critical research and clinical priority.

Rigorous evaluation of the effectiveness of interventions depends upon robust outcome measurement (10, 11). One widely used measure of behavioural and emotional problems amongst children with intellectual disabilities is the Developmental Behaviour Checklist, parent report (DBC-P) (12). The DBC-P was also updated to form the DBC2-P but, given the changes were minor, we use DBC to refer to both measures unless otherwise specified. Evidence has indicated strong psychometric properties for the DBC-P such as its validity, reliability, and responsiveness to change (12). However, evaluating an intervention’s effectiveness also relies upon an understanding of what changes in scores on an outcome measure such as the DBC-P are sufficient to be considered clinically or otherwise meaningful. This relates to the concept of a Minimum Clinically Important Difference (MCID) - the smallest change in an outcome measure that is important to those receiving an intervention and/or other stakeholders (13, 14).

There are several reasons that estimating a MCID for an outcome measure is important. With sufficiently large samples, clinically negligible intervention effects may be statistically significant – making statistical significance alone a poor standard by which to judge intervention effectiveness. MCID estimates offer a valuable standard for evaluating whether intervention effects are sufficient to be considered meaningful, as well as statistically robust (13, 14). Second, an MCID may be used to inform sample size calculations to ensure that studies are appropriately statistically powered to identify meaningful effects (15, 16). Finally, an MCID can be directly used by clinicians and service providers to evaluate individual treatment outcomes against a meaningful standard and inform clinical decision-making (14).

There are two broad categories of approaches to estimating an MCID (13, 14, 17). Distribution-based methods are based on the statistical characteristics of studies, such as the change in scores that correspond to a chosen effect size cut-off in a sample (13, 14). Anchor-based methods compare a change in scores with an external criterion, often by identifying changes in the scores of participants who describe themselves as having improved (vs not) following an intervention (13, 14). However, it has also been suggested that an MCID could be anchored to stakeholders’ own direct estimates of what change in scores they would consider to be important or meaningful (17). Indeed, direct consultation with parent carers and professionals has been used to estimate an MCID (18) for the Aberrant Behaviour Checklist – Irritability sub-scale (19).

Although these two approaches are widely used (13, 14), both have limitations. Distribution-based approaches are criticised for the arbitrariness of chosen effect size cut-offs and the lack of grounding in stakeholder perspectives, whilst anchor-based approaches have been criticised for their susceptibility to influence by recall bias and individuals’ current health states (14). A valuable approach is, therefore, to adopt both distribution-based and anchor-based methods in conjunction to generate informative MCID estimates (14, 17, 18).

The research question for the current study was: What is a suitable Minimum Clinically Important Difference for the Total Behaviour Problem Score of the Developmental Behaviour Checklist (DBC2), parent carer report-form?

## Methods

This study was preregistered before collecting any data or conducting the literature searches (https://osf.io/ctwum/?view_only=c08ab197ce654e608dade4c451df22f0). Data for the meta-analyses and R scripts have also been deposited for transparency (https://osf.io/v6kud/files/osfstorage). The distribution-based MCID estimates were generated through a systematic review and meta-analysis, whilst the anchor-based estimates were generated through consultation with parent carers and healthcare professionals.

### Distribution-based approach

To generate distribution-based estimates for an MCID on the DBC-P Total Behaviour Problem Score, we conducted a systematic review and meta-analysis of intervention evaluation studies that included the DBC-P as an outcome measure.

#### Eligibility criteria

##### Population

Participants needed to have an intellectual disability, autism, or a genetic syndrome associated with intellectual disability and/or autism. Intellectual disabilities, autism, or associated genetic syndromes could be confirmed by report of a diagnosis by a family member, receipt of intellectual disability education or services, genetic testing, or meeting diagnostic thresholds on psychometric tests within the study. Participants may have had other additional diagnoses as well as intellectual disability, autism, or an associated genetic syndrome. Studies were eligible if data were reported for a group in which ≥70% of participants met this criterion. Participants were aged between 4 and 18 years as this is the age range for the DBC-P. Studies were eligible if data were reported for a group in which ≥70% of participants meet this criterion. Studies were excluded if participants had a specific learning difficulty or other neurodevelopmental condition (e.g. dyslexia, dyscalculia, ADHD) but did not have an identified intellectual disability, autism, or an associated genetic syndrome.

##### Intervention

Any intervention study which used the DBC-P, parent version as a primary or secondary outcome measure was eligible, irrespective of the nature of the intervention.

##### Types of study

Randomised controlled trials, non-randomised controlled trials, and uncontrolled pre-post studies were eligible. We excluded case series and case studies, including single-case experimental designs.

##### Comparator

Studies with any control comparison or none were eligible.

##### Context

Any context of intervention delivery was eligible for inclusion

##### Outcomes

The Developmental Behaviour Checklist, parent-form or Developmental Behaviour Checklist 2, parent form, including authorised translations of the DBC2-P in languages other than English. We excluded studies which reported only on other versions of the DBC-P such as the teacher version, the adult version, the short form, or individual items, subscales, or subsets of items of the DBC but not information required to calculate the Total Behaviour Problems Score.

#### Search strategy

We searched Medline, Embase, PsycINFO, and Web of Science (all databases) using the terms “Developmental Behaviour Checklist” OR “Developmental Behavior Checklist”. The last searches took place on 22/08/2024 and there were no date restrictions. Using Google Scholar and Web of Science, we also conducted forward citation searches of key DBC-P psychometric publications (12, 20–23). Finally, once eligible full texts had been identified, forwards and backwards citation searches were conducted on these to identify any other eligible research.

#### Study selection

All identified records were then imported into Covidence (24) and underwent electronic de-duplication. All of the titles and abstracts of the remaining records were independently screened by two reviewers (DS and ET) in Covidence, with discrepancies being discussed and a consensus reached. During title and abstract screening, the reviewers showed a good level of agreement (96.44%, Kappa = 0.702). All remaining records then underwent independent full-text screening by two reviewers, with discrepancies being resolved through discussion. A good level of agreement for inclusion during full-text screening was reached (agreement= 88.57%, Kappa= 0.701).

#### Data extraction

Data were extracted independently by two reviewers (DS and ET) from 100% of eligible studies. Discrepancies were discussed between the two reviewers and an agreement was reached. If data necessary for meta-analysis were not available, study authors were contacted to request this information.

#### Analysis

We calculated the pooled SD from the baseline intervention groups of all studies to generate estimates for a change score on the DBC-P for small (d=0.2), medium (d=0.3), and large (d=0.5) effect sizes.

We then conducted random-effects meta-analyses of DBC-P scores based on: 1) ANCOVA estimates for RCTs; 2) final outcome scores for RCTs; and 3) pre-post changes in DBC-P scores for controlled and pre-post studies. The original pre-registration proposed meta-analysing the change in DBC-P scores for all study designs together, after adjusting for the change in control comparisons where one was present. However, we judged that separate analyses of RCTs and pre-post changes would provide more informative estimates of potential MCIDs for each study design. The pre-registration outlined that meta-analyses would be conducted using the R package *metafor* (25). However, for the analyses of RCTs, we used the R shiny tool by Papadimitropoulou et al. (26) since this is largely powered by *metafor* but offers more options for ANCOVA analyses which better account for baseline differences between trial arms. Since the correlations between baseline and follow-up DBC-P scores which are required for ANCOVA estimates could only be obtained from 9/15 trials, with the remaining correlations being automatically imputed, we also conducted a meta-analysis of RCTs comparing final outcome scores as a more conservative estimate. Several crossover RCTs were identified in the study selection process. Including all data from these crossover trials in the meta-analysis as though they were parallel group trials would give rise to a unit-of-analysis-error and provide overly conservative effect estimates (27). In two trials where data were reported for each group at each timepoint (28, 29), we therefore used only data from the first intervention that participants received (27). However, one trial (30) did not report data necessary for this approach and so we evaluated the effect of including/excluding this study in the meta-analyses in **Supplementary Materials B**. For one study (31) which compared two active intervention arms, both of the two arms were analysed separately in the meta-analysis of pre-post studies.

If the purpose of the meta-analyses were to evaluate the effectiveness of interventions, it would be important to investigate potential sources of heterogeneity in effect sizes. However, since the analyses are focused on exploring the range of outcomes in studies using the DBC, such examinations of heterogeneity were judged not relevant to the current research and were not conducted.

### Anchor-based approach

We generated MCID estimates which were anchored to the views of parent carers and professionals through consultation. We developed five clinical vignettes (see **Supplementary Materials A**) describing children and adolescents with varying severity and range of behavioural and emotional problems. Four of these vignettes were adapted from examples in the DBC2 manual and one was entirely new. The vignettes were deliberately designed to reflect varying severity of intellectual disability, DBC total scores, and presentations of behavioural and emotional problems. Parent carers and professionals were presented with these cases and the individual’s current DBC-P scores before being asked to identify the smallest possible change in DBC-P Total Behaviour Problem Score that would be needed for them to consider an intervention to have had a meaningful beneficial effect for that individual and their family. Participants were asked to provide this judgement both through indicating which individual items on the DBC-P would be important to change and by what amount to be meaningful, and what the minimum meaningful change would be overall across those items. These data were collected from 10 parent carers, who were part of existing co-production research groups, through individual online meetings. Meanwhile, data were collected from 21 health and social care professionals: six through direct consultation meetings and 15 through survey responses. The professionals were recruited through professional networks of the research team, social media, and through distributing the survey through a professional mailing list for clinicians and researchers working with people with an intellectual disability in the United Kingdom. There were initially 18 completed survey responses, but two were excluded because the respondents subtracted one point from every item, and so we judged that it was not clear they had engaged with the task instructions; and one further response was excluded because they were a General Practitioner and did not report working in a role with a particular focus on children or adolescents with intellectual disabilities. The 21 professionals included 12 psychologists, 3 psychiatrists, 2 speech and language therapists, 1 nursing manager, 2 behaviour analysts, and 1 Positive Behaviour Support practitioner. The pre-registration initially stated that we would report separate MCID estimates for case vignettes with low, moderate, and severe levels of behavioural and emotional problems. However, we decided to report only one combined MCID estimate based upon all cases given the small sample size.

## Results

**Figure 1:**
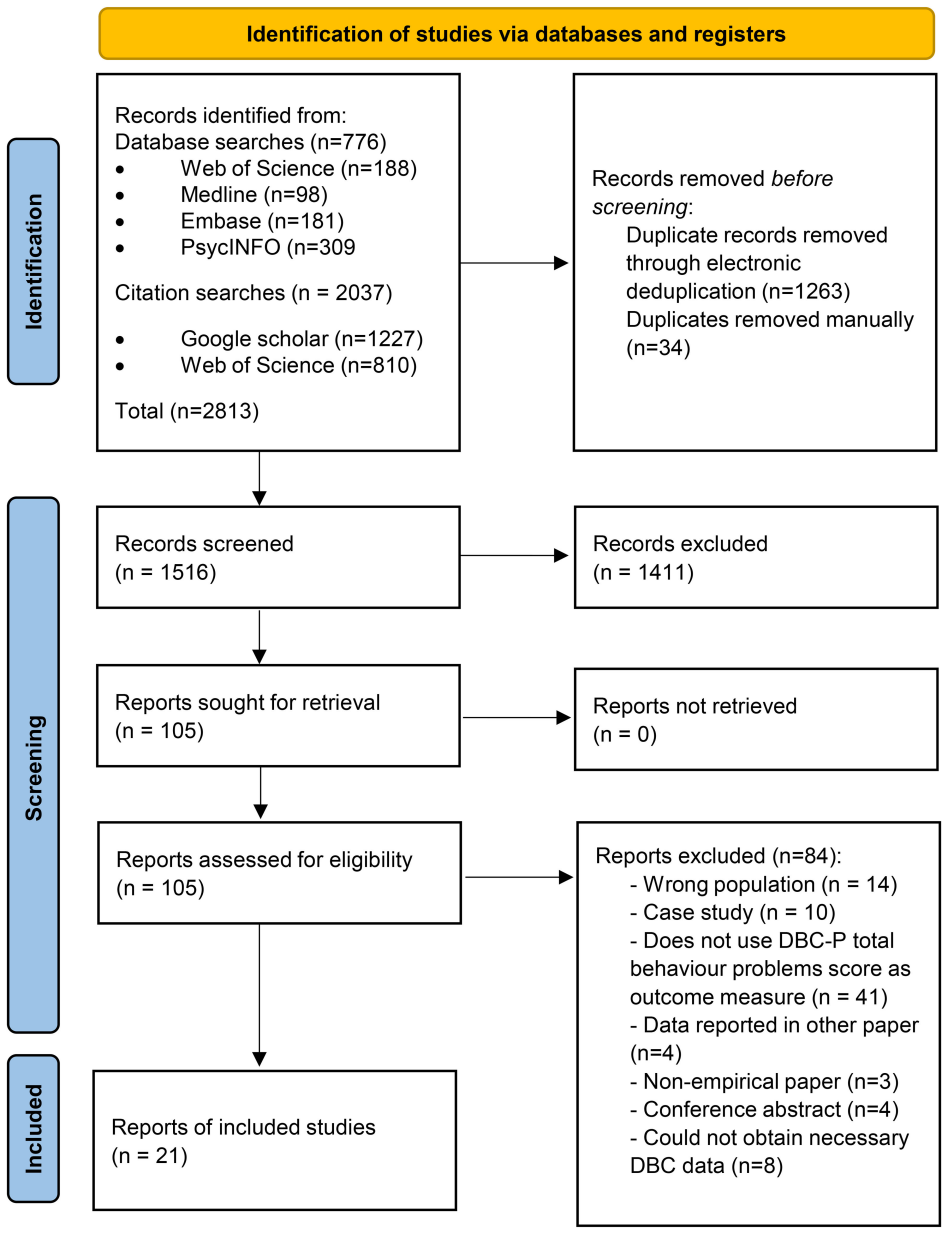
PRISMA diagram (32) illustrating the search and study selection process.

**Figure 1** shows a PRISMA diagram (32) illustrating the search and study selection process for the meta-analysis. We identified 776 records through database searches and 2037 records through forwards and backwards searches of the key DBC-P references. 1263 records were removed by electronic de-duplication in Covidence and 34 duplicates were removed manually. Of 1516 records which underwent title and abstract screening, 1411 were excluded, leaving 105 to undergo full-text screening; and 84 of these were excluded at full-text screening, leaving 21 records included in the review.

### Study characteristics

**Table 1** summarises the characteristics of each study. Included studies were mostly journal articles (n=17) except for three theses (33–35) and one pre-print (28). Studies were mostly conducted in Australia (n=15) (28, 29, 31, 33–44), where the DBC-P was developed, along with small numbers in the UK (n=3) (30, 45, 46), Germany (n=2) (47, 48), and Greece (n=1) (49). Study designs included 12 randomised controlled trials (31, 33, 35–41, 43, 44, 47), three crossover randomised controlled trials (28–30), five pre-post studies (34, 45, 46, 48, 49), and one non-randomised controlled trial (42). Although many studies included participants who were autistic and had intellectual disabilities, 14 (28–31, 33, 35–39, 44, 46, 48, 49) studies had samples where all participants were autistic, three had samples where all participants had intellectual disabilities (43, 45, 47), and four recruited samples of participants who were either autistic or had intellectual disabilities, or where all participants had both conditions (34, 40–42).

**Table 1:** Characteristics of studies included in meta-analysis.

Author	Country	Study design	Participant age	Participants’ diagnoses	Sample size	Intervention	DBC-P primary or secondary outcome
Bettison, 1996 (36)	Australia	RCT	Range=3.9 to 17.1 years.	Autism	N=80 Intervention (n=40) Control (n=40)	Auditory integration training versus structured listening. Two 30-minutes listening sessions, twice daily for ten days.	Unspecified
Dean et al., 2017 (37)	Australia	RCT	Placebo: M = 6.24, SD = 1.98 Intervention: M= 6.53, SD = 1.81 Range = 3.1–9.9 years	Autism, with/without ID. Intervention: FSIQ: M(SD)=71.7(13.8) VABS ABC M(SD)=69.9(12.5) Control: FSIQ M(SD)= 74.3(15.6) VABS ABC M (SD)=70.7(14.7)	Baseline: N= 98 Intervention (n=50) Control (n=48) Follow up: N=71 Intervention: (n=37) Control: (n=34)	500mg/day oral N-acetyl cysteine versus placebo capsules	Secondary
Elliott, 2017 (35)	Australia	RCT	Intervention: M = 8.85, SD = 1.77, range = 6–11 years Waitlist: M = 8.25, SD = 2.19, Range = 6–11 years	Autism	N = 21 Waitlist (n=8) Intervention (n=13)	Whiz Kid Games - online independent living and social skills intervention	Primary
Guastella et al., 2023 (38)	Australia	RCT	Oxytocin: Mean=7.40, SD=2.95, Range=3.11–12.81 Placebo: Mean**=**7.12, SD=2.42, Range=3.20–12.58	Autism + 12% with ID Intervention: FSIQ M (SD)= 95.47(16.12) Placebo: FSIQ M(SD)=98.00(14.98)	N=97 randomised Oxytocin (n=49) Placebo (n=48) N=87 analysed Oxytocin (n=45) Placebo (n=42)	Oxytocin nasal spray (32 international units per day) versus placebo spray.	Secondary
Guastella et al., 2015 (39)	Australia	RCT	Intervention: M=13.85, SD=1.54 Control: Mean=14.00, SD=2.04 Range =12–18 years	Autism Intervention FSIQ: M=80.04, SD=19.18 Control FSIQ: M=93.14, SD=21.11	N=50 randomised and analysed Intervention (n=26) Placebo (n=24)	Oxytocin nasal spray (36–48 international units per day) versus placebo spray.	Secondary
Hinton et al., 2017 (40)	Australia	RCT	Intervention: M=6.33, SD=2.43 Control: M=5.66, SD=2.15 Range = 2–12 years	Heterogenous including autism, genetic syndromes, learning disability, acquired brain injury, physical disabilities, sensory disabilities.	N= 98 randomised Intervention (n=51) Control (n=47) Post-intervention N= 89 Intervention (n=46) Control (n=43) Follow-up (intervention only) n=38	Triple P Online-disability - telehealth intervention combining TPOL and SSTP elements versus treatment as usual	Unspecified
Kasperzack et al., 2020 (48)	Germany	pre-post	M=7.12, SD=2.78, range=3.6-12	Autism + 33.3% with ID	24	Group SSTP (behavioural parenting intervention)	Primary
Kostulski et al., 2021 (47)	Germany	RCT	Intervention: M = 10.89 SD = 2.90 Waitlist: M = 11.23 SD = 2.73 Range = 6–16 years	Intellectual disability- 76% moderate and 26% mild	N = 42 randomised Intervention (n=21) Waitlist (n=21) Follow-up N=35 Intervention n = 17 Waitlist n=18	Group parent management training. Ten 90-minute sessions over 6 months	Primary
Makrygianni & Reed, 2010 (49)	Greece	pre-post	M= 6.63 (SD = 3.11)	Autism or PDD-NOS IQ M(SD)= 64.86(28.08) VABS ABC M(SD)=48.06(18.81)	86	School-based intervention programmes with little description	Unspecified
Moss et al., 2014 (41)	Australia	RCT	M = 11.74 (SD = 2.53), range 8-17	Autism (n=15), genetic syndrome (n=5), intellectual disability (n=3), blindness (n=1), unspecified (n=2)	N=26 Intervention n=13 Control n=13 n=22 completed post-treatment and n=18 completed follow-up measures	The Sleepwise program (manualised behavioural sleep intervention) versus waitlist control	Secondary
Mulligan et al., 2015 (45)	UK	pre-post	M=9.11, SD=3.95, Range=2-17	Intellectual disability	Total N=45 Analytic sample n=38	Family Intensive Support Service. Multicomponent, intensive intervention.	Unspecified
Parrella et al., 2024 (28)	Australia	crossover RCT	M = 9.62 years, SD = 2.05, Range = 5-12	Autism + 32.35% with intellectual disability	N=34 but 5 dropped out during intervention period leaving sample of n=29	Cannabidiol (CBD) oil (10 mg/kg/day) versus placebo oil	Secondary
Pillay et al., 2021 (46)	UK	pre-post	M and SD not reported. 55% aged 4-8, 29% aged 9-13, 16% aged 14-18	Autism	Initial sample of N=58. n=35 completed pre and post DBC-P s	ASCEND- Manualised group psychoeducational and peer support.	Unspecified
Ratcliffe et al., 2019 (42)	Australia	non-randomised controlled trial	Treatment: M**=**9.41, SD=1.48 Waitlist: M= 9.12, SD=1.36	Autism and mild intellectual disability	Total n=75 Baseline Intervention (n=37) Control (n=24) Post Intervention (n=26) Control (n=14)	EBSST. 16 group sessions delivered by school counsellors. Compared to waitlist.	Unspecified
Roberts et al., 2006 (43)	Australia	RCT	Intervention: M=4.42 SD=0.92 Control: M=4.21 SD=1.08	“Developmental disabilities with levels of intellectual or adaptive functioning two or more standard deviations below the mean”. Intervention: IQ M=61.19, SD=14.64 Control: M=63.79, SD=18.58	N= 48 Intervention (n=27) Control (n=20)”	SSTP	Unspecified
Roberts et al., 2011 (31)	Australia	RCT	Home-based group: M=41.5, Range=26.5–59.4 Centre-based group: M=43.12, Range=6.3–60.0	Autism Mean Griffiths Developmental Quotient Home-based group: M=57.0 SD = 11.7 Centre-based group: M=66.5 SD=17.7	N=67 randomised Outcome data available on: Home-based intervention (n=27) Centre-based intervention (n=29)	Building blocks (multicomponent early intervention), delivered as a centre-based group programme, versus as a home-based programme.	Primary
Roux et al., 2013 (44)	Australia	RCT	Combined: M=4.8, SD=1.64 Treatment: M=4.81, SD =1.5 Waitlist: M=4.6, SD=1.9	Autism (with and without ID), Down syndrome, Cerebral Palsy, or intellectual disability	N = 55 randomised Waitlist (n=27) Intervention (n=28) Analysed: Intervention Pre-post intervention (n = 28) Post intervention-follow up (n = 24) Waitlist: Pre-post (n=27)	Group SSTP (behavioural parenting intervention)	Unspecified
Walsh, 2008 (34)	Australia	pre-post	M=5.88, eligibility ranged from 1.5-15	30.7% autism/PDD-NOS, 21.3% developmental delay, 17.3% intellectual delay	Total N=79 Analysed (n=67)	Group SSTP (behavioural parenting intervention)	Unspecified
Williams, 2017 (33)	Australia	RCT	Intervention: M (months): 62.83 SD = 11.17 Range = 48.20-84.24 Control: M (months): 61.93 SD = 9.91 Range = 48.10 - 83.09	Autism, 29% of participants had IQ scores within ID range. Intervention: FSIQ M(SD)= 77.93(13.96) VABS ABC M(SD)= 73.41(12.21) Control: FSIQ M(SD) = 74.56(13.58) VABS ABC M(SD) =73.48(9.97)	N=60 randomised DBC-P data on 31 Intervention (n=18) Control (n=13)	Transporters emotion recognition training DVD series vs control DVD	Unspecified
Wright et al., 2011 (30)	UK	crossover RCT	Range = 4–16 years Melatonin first: M_age_ = 8.9 years, SD = 3.0 Placebo first: M_age_ = 8.5 years, SD = 2.3	Autism	N = 20 randomised Melatonin first (n=9) Placebo first (n=11) N=16 data for analysis Melatonin first (n=6) Placebo first (n=10)	Titrated 2-10mg standard release melatonin versus placebo	Secondary
Yatawara et al., 2016 (29)	Australia	crossover RCT	Oxytocin then Placebo: M = 5.7, SD = 1.5, range = 3.1 - 8.0 Placebo then oxytocin: M = 6.7, SD = 1.8, range = 3.0 - 8.9	Autism/PDD-NOS Oxytocin then placebo: NVIQ: M(SD)=76.4(22.9) Placebo then oxytocin: NVIQ= M(SD) = 91.9(23.7)	N= 39 Oxytocin then placebo (n=17) Placebo then oxytocin (n=22) N = 31 analysed	Oxytocin nasal spray (24 international units per day) versus placebo	Secondary

ASCEND, Autism Spectrum Conditions – Enhancing Nurture and Development; EBSST, Emotion-Based Social Skills Training; FSIQ, Full Scale Intelligence Quotient; M, Mean; NVIQ, Non-Verbal Intelligence Quotient; PDD-NOS, Pervasive Developmental Disorder – Not Otherwise Specified; RCT, Randomised-controlled trial; SD, Standard Deviation; SSTP, Stepping Stones Triple P; TPOL, Triple P Online; VABS ABC, Vineland Adaptive Behaviour Scales Adaptive Behaviour Composite.

### Interventions

Studies included both pharmacological (n=6) and non-pharmacological interventions (n=15). Pharmacological interventions included oxytocin nasal spray (n=3) (29, 38, 39), melatonin (n=1) (30), cannabidiol (CBD) oil (n=1) (28), and N-acetyl cysteine (n=1) (37). The most common non-pharmacological interventions were Triple P behavioural parent training programmes (n=5). Three of these were evaluations of group Stepping Stones Triple P (the version of Triple P for parents of disabled children) (34, 44, 48), one was an evaluation of individual Stepping Stones Triple P (43), and one evaluated Triple P Online – Disability (TPOL-D) an adaptation of Triple P Online for parents of disabled children (40). Several studies (n=3) evaluated social skills interventions. These included Transporters (a DVD-based emotion-recognition training programme) (33), Emotion-Based Social Skills Training (EBSST) – a group programme delivered by school counsellors (42), and Whiz Kid, an online videogame-based intervention (35). Two studies evaluated group parent support programmes besides Triple P. These were Autism Spectrum Conditions – Enhancing Nurture and Development (ASCEND), (a group-based psychoeducational programme) (46), and group parent management training (47). The remaining studies evaluated varied interventions including a manualised behavioural and psychoeducational sleep intervention (41), a family intensive support service (45), auditory integration training (36), ambiguous school-based interventions (49), and Building Blocks, a multicomponent autism early intervention programme (31).

### Pooled standard deviation

The pooled SD of the baseline intervention groups of all studies was 23.71 but ranged in individual studies from 14.82 to 32.91. Based upon the pooled standard deviation, changes in DBC-P scores for small (d=0.2), medium (d=0.3), and large (d=0.5) effect sizes would be approximately 4.74, 7.11, and 11.86, respectively.

### Meta-analysis of RCTs

#### ANCOVA meta-analysis of randomised controlled trials

**Figure 2:**
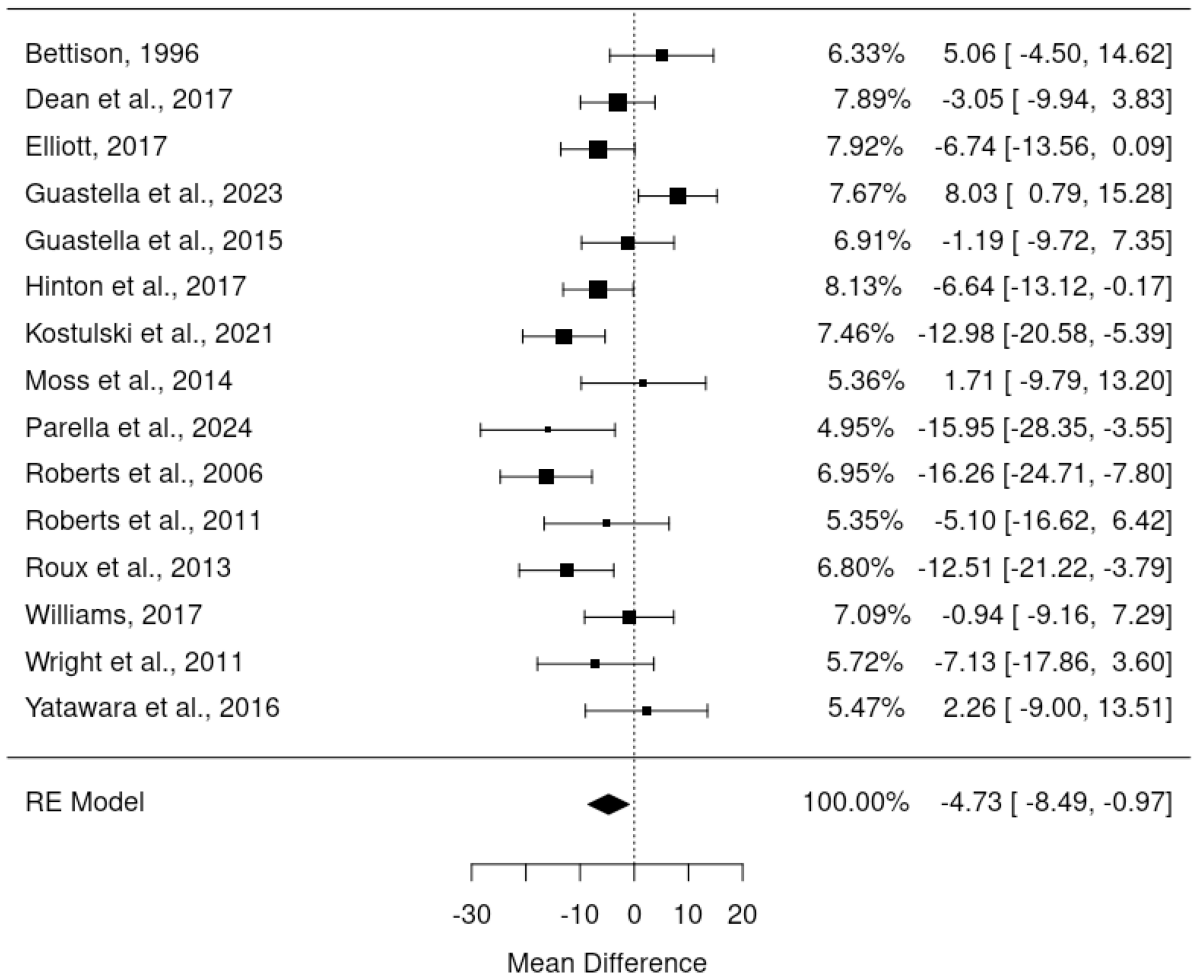
Forest plot illustrating random effects ANCOVA meta-analysis of RCTs.

**Figure 3:**
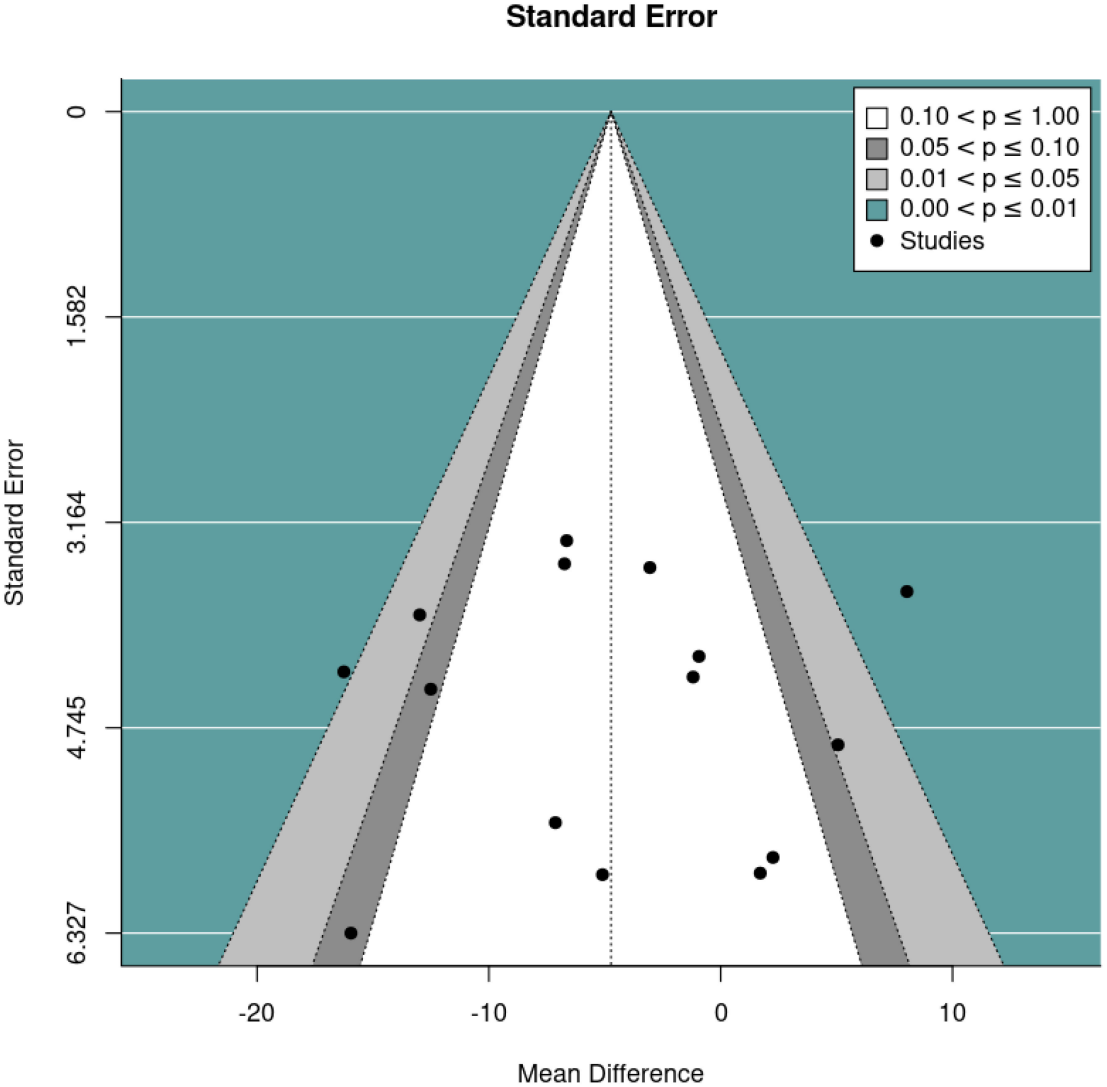
Funnel plot for the random effects ANCOVA meta-analysis of RCTs.

The primary meta-analysis was a random-effects model which adjusted for baseline DBC-P scores based on ANCOVA recovered effect estimates. This provided an estimated mean difference of -4.73, standard error= 1.92, 95% CI= -8.49, –0.97, p=0.014, k=15, tau^2^ = 34.42, I^2^ = 64.40%. The large I^2^ suggests considerable heterogeneity; yet this is relatively unsurprising given the diversity of the interventions and participant samples included. **Figures 2** and **3** show a forest plot and funnel plot respectively for the ANCOVA meta-analysis of RCTs. Removing the crossover trial without complete data at each timepoint (30) resulted in a slightly smaller estimated mean difference of -4.59 but did not substantially alter the overall findings (see **Supplementary Materials B**). Visual inspection of the funnel plot suggests approximate symmetry and, therefore, little evidence of publication bias.

#### Meta-analysis of randomised controlled trials final outcome scores

**Figure 4:**
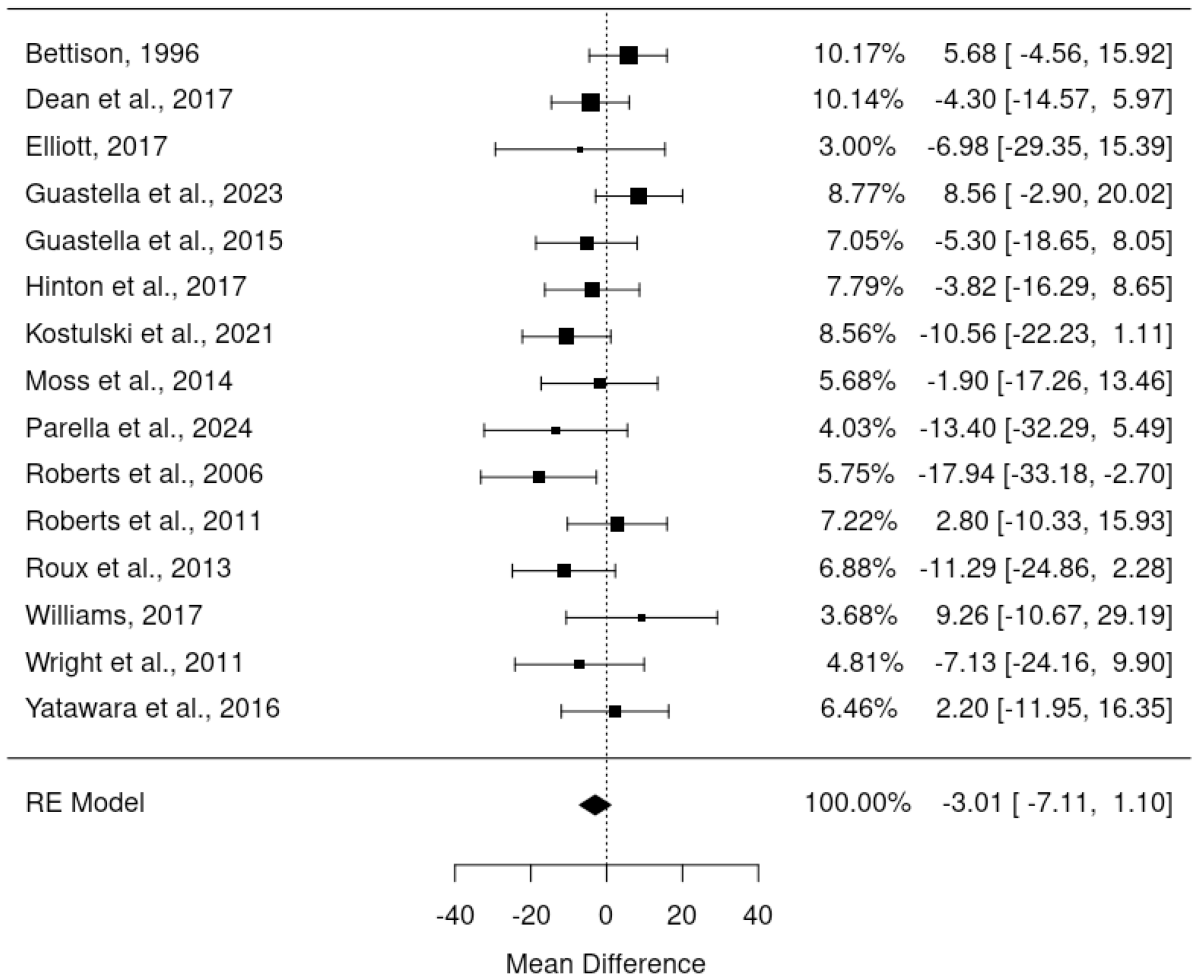
Forest plot illustrating random effects meta-analysis of final scores in RCTs.

**Figure 5:**
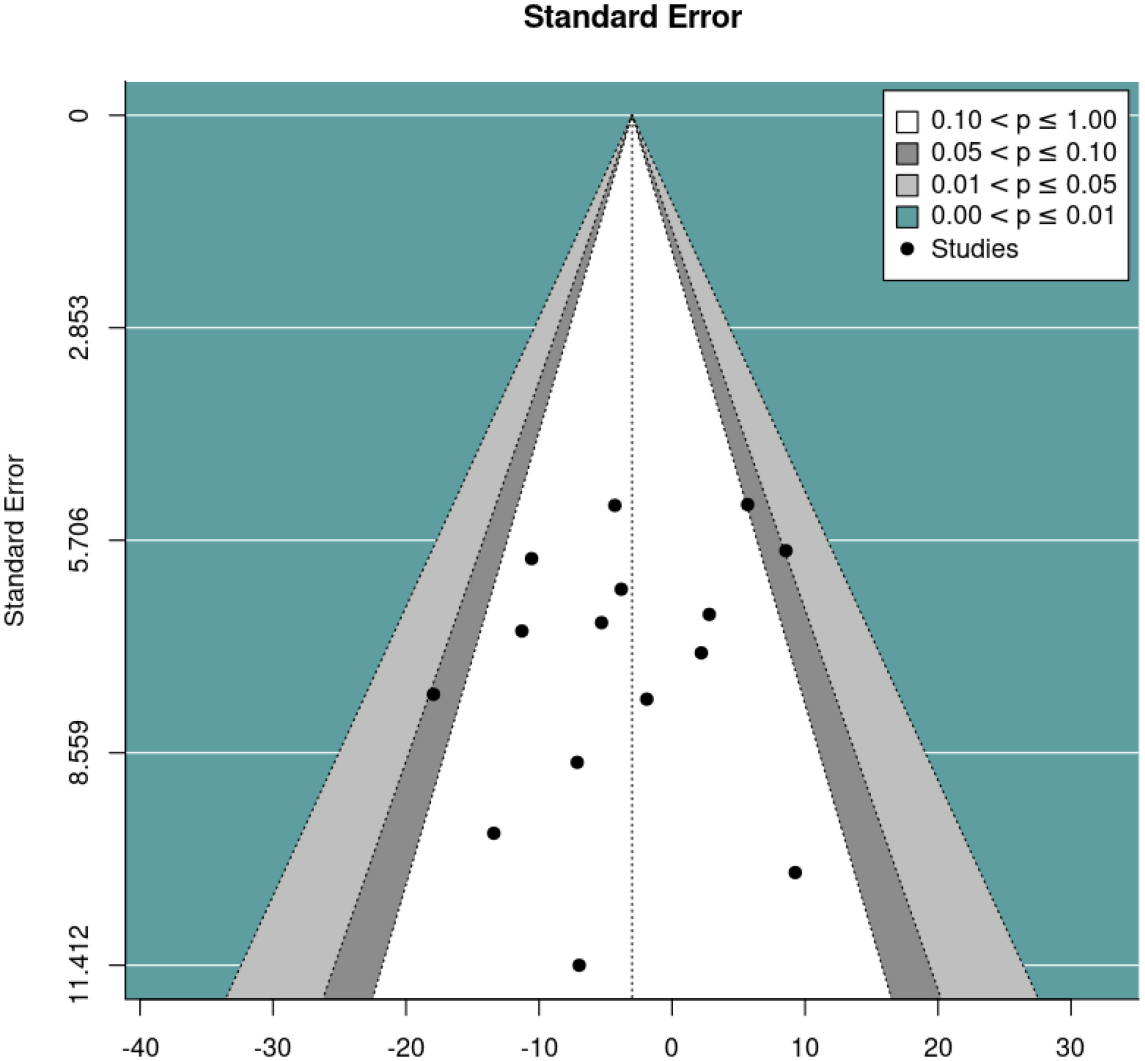
Funnel plot for the random effects meta-analysis of final scores in RCTs.

Since pre-post DBC-P correlations which are required for ANCOVA were not available for all RCTs, we also conducted a random-effects meta-analysis of change scores. This provided an estimated mean difference of -3.01, standard error= 2.09, 95% CI= -7.11, 1.10, p=0.15, k=15, tau^2^ = 15.82, I^2^ = 24.61%. **Figures 4** and **5** show forest and funnel plots respectively for the meta-analysis of RCT change scores. Removing the crossover trial without complete data at each timepoint (30) resulted in a slightly smaller estimated mean difference of -2.82 but did not substantially alter the overall findings (see **Supplementary Materials B**). Visual inspection of the funnel plot indicated possible asymmetry, with fewer studies with higher standard error showing reductions in DBC scores, which could indicate publication bias. However, this is difficult to evaluate with confidence given the small number of studies.

#### Meta-analysis of pre-post DBC-P change

**Figure 6:**
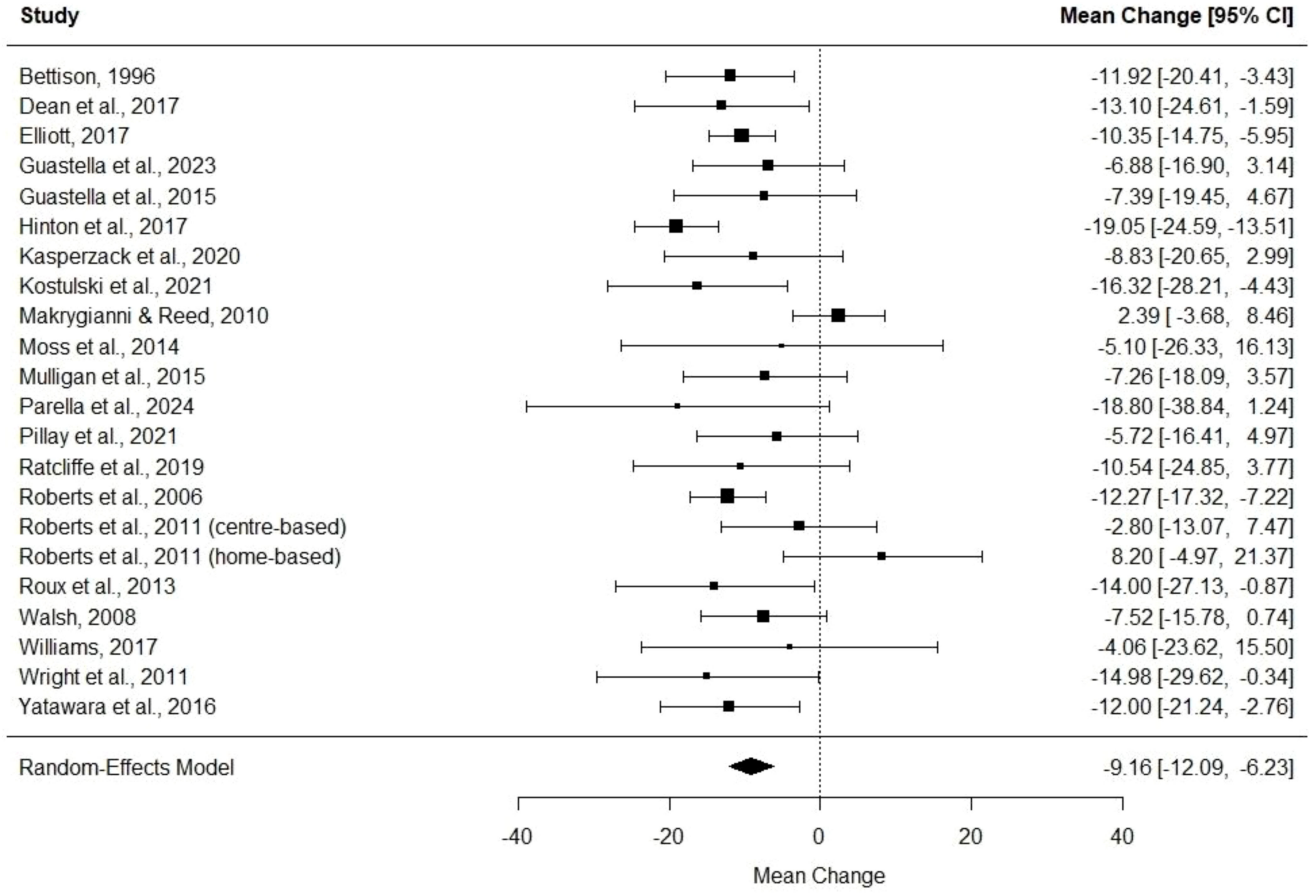
Forest plot illustrating random effects meta-analysis of pre-post change in DBC-P scores.

**Figure 7:**
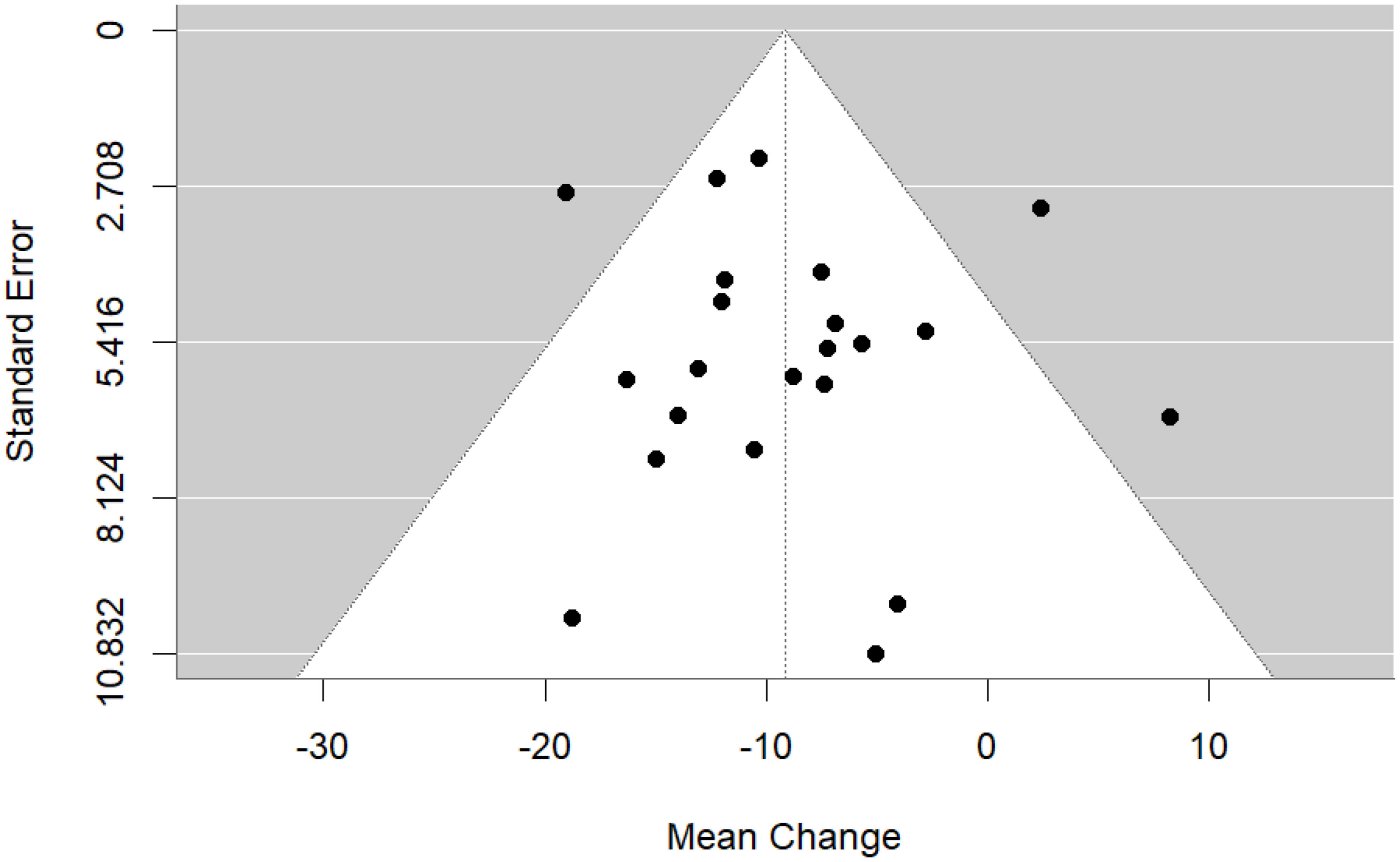
Funnel plot for the random effects meta-analysis of pre-post change in DBC-P scores.

We then performed a random effects meta-analysis of pre-post studies. This indicated a mean difference of -9.16, standard error= 1.49, 95% CI= -12.09, -6.23, p<0.001, k=22, tau^2^ = 20.82, I^2^ = 49.69%. **Figures 6** and **7** show forest and funnel plots respectively illustrating the results from the meta-analysis of pre-post changes in DBC-P scores Visual inspection of the funnel plot indicated a possible small amount of skew, which could suggest some publication bias, but this is difficult to evaluate with confidence given the small number of studies.

### Anchor-based approach

#### Consultation with parent carers

**Figure 8:**
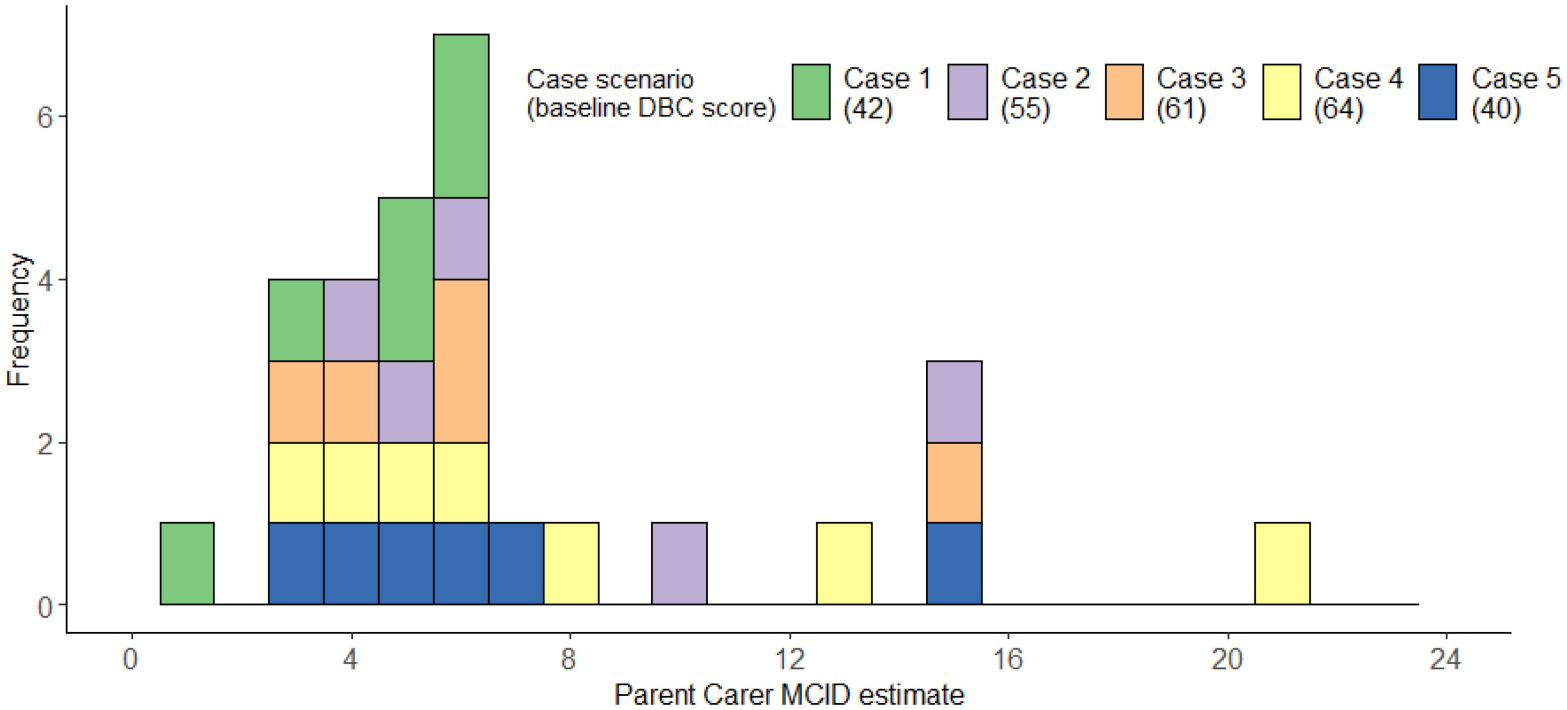
Histogram illustrating parent carers’ MCID estimates for each case scenario.

The 10 parent carers we consulted completed an average of 2.90 case scenarios (range = 2-4). The median MCID across scenarios was 6, IQR=4-7; range=1-21, mean=6.90, bootstrapped, bias-corrected 95% confidence intervals (1000 repeats) = 5.55-8.93, SD=4.62 (**Figure 8**). **Figure 8** illustrates that parent carer estimates clustered around 3–6 points, with smaller numbers of substantially higher estimates. Descriptive statistics for each individual case can be found in **Supplementary Materials C**.

#### Consultation with professionals

**Figure 9:**
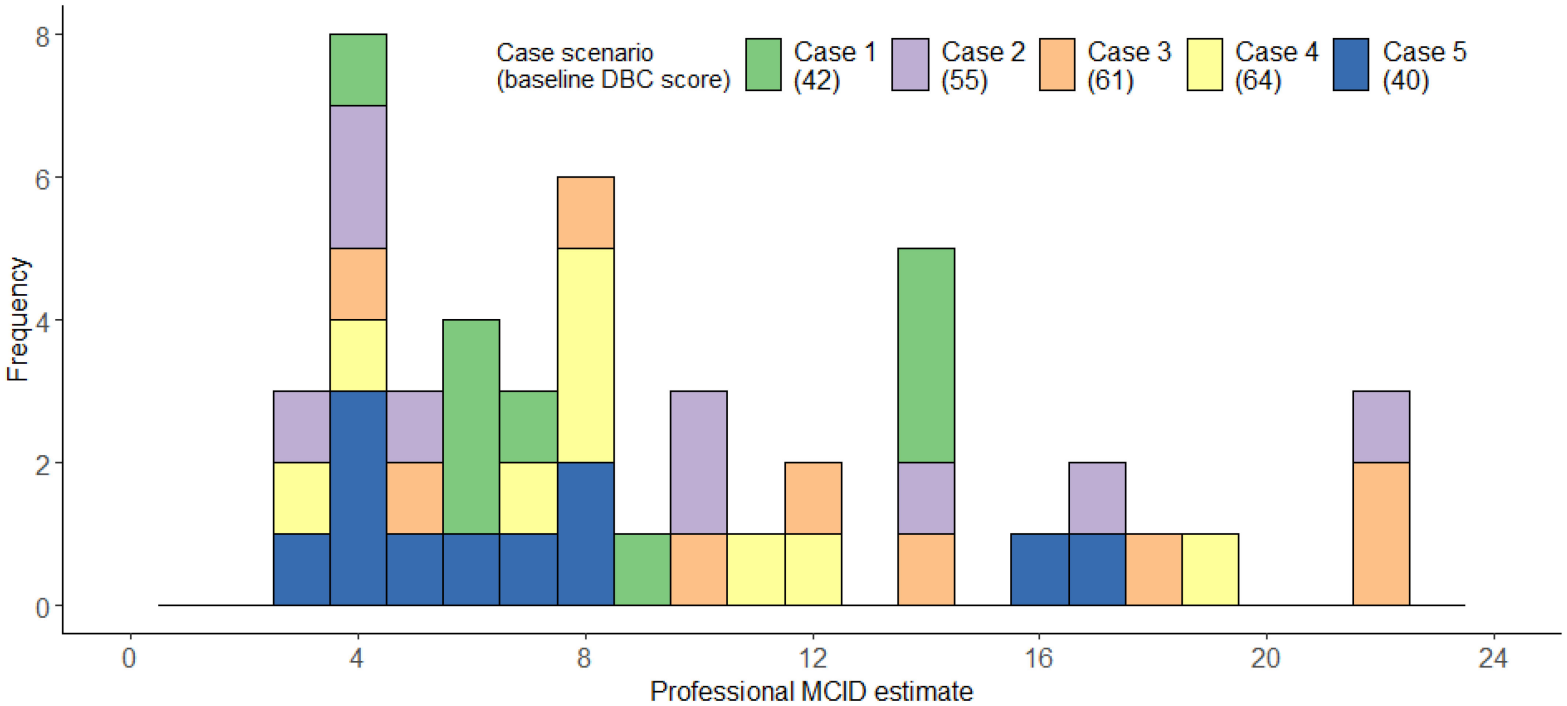
Histogram illustrating professionals’ MCID estimates for each case scenario.

The 21 professionals completed an average of 2.24 cases each, providing a total of 47 MCID estimates. Professionals’ median MCID across scenarios was 8, IQR=5-14; range=3-22, mean=9.49, bootstrapped, bias-corrected 95% confidence intervals (1000 repeats) =8.06-11.24, SD=5.54 (**Figure 9**). **Figure 9** illustrates that professionals’ MCID estimates were more variable- with some possible clustering around 3–8 points of change, but many estimates being much higher. Descriptive statistics for each individual case can be found in **Supplementary Materials C**.

## Discussion

We describe an approach to estimating an MCID for the DBC-P Total Behaviour Problems Score through both distribution-based and anchor-based methods. Meta-analyses indicated an average decrease of approximately 3.01 to 4.73 points in RCTs, depending on analytic methods, with a larger average decrease of 9.16 in pre-post studies as would be expected. This overlaps with parent carers’ anchor-based estimates – with 68.97% of parent carers MCID estimates being between 3 and 6 points. Professionals’ MCID estimates showed greater variability, with some professionals providing larger estimates, but 38.30% of estimates were still between 3 and 6 and the modal estimate was still a 4-point decrease. What constitutes a meaningful change in a specific context may be influenced by factors such as an individual’s baseline behavioural and emotional problems score, the nature and intensity of an intervention, and the context in which the child or young person is being assessed. However, where standardised MCID estimates may be valuable, such as for trial sample size calculations, our findings suggest that an appropriate MCID may be in the region of approximately 3–6 points. Based upon the pooled SD from studies included in the meta-analyses, this would correspond to a standardised mean difference of between -0.13 and -0.25.

It is notable that professionals’ MCID estimates were, on average, approximately 2–3 points larger than those of parents. This should be interpreted with considerable caution given the small sample sizes and variable estimates provided. However, it could indicate that, compared to professionals, parents consider smaller decreases in behavioural and emotional problems to be meaningful perhaps because they are coping with these challenges on a daily basis and even small improvements may lead to considerable positive impact for the family. Professionals may benefit from reflecting on the potential importance to parents of seemingly small changes in behavioural and emotional problems when considering how best to support families. Alternatively, the higher scores among professionals may simply be driven by greater variability in estimates, which could be related to factors such as clinical discipline. Unfortunately, the sample of professionals in this research is too small to allow comparisons between different groups of healthcare professionals.

These MCID estimates should not be viewed as some intrinsic property of the DBC-P, nor uncritically adopted by researchers or clinicians without careful consideration about how they were generated and how this relates to the intended use. Research has demonstrated that MCID estimates generated using different methods are frequently non-convergent (14, 17). MCID estimates though are still useful. Regardless of the complexity of their estimation, researchers, clinicians, and policymakers cannot avoid decisions that rely upon consideration of what constitutes an MCID; for example, when designing RCTs, evaluating patient outcomes, or deciding whether to fund an intervention. Our goal was to provide readers with data regarding the views of stakeholders and the approximate changes in DBC-P scores that may reasonably be anticipated from a broad range of interventions. We hope that this may allow readers to make well-informed and empirically grounded judgements of their own.

Several strengths of this research should be noted. Integrating distribution-based and anchor-based methods from both parent carers and professionals provides a multifaceted understanding of appropriate MCIDs for the DBC-P and allows researchers to prioritise these according to their own circumstances and priorities (14, 17). However, there are also several limitations to this work. We only obtained data from a small number of parent carers, which may impact the reliability and generalisability of the estimate, and the larger variability in professionals’ estimates suggests a lack of consensus in what constitutes an MCID. Another challenge in generating MCID estimates was the perceived non-equivalence of individual DBC-P items. Parents and professionals would frequently comment that a decrease of a single point in one critical item (often relating to self-injury, aggression, school non-attendance, or running away) could be more meaningful than a larger decrease for several items perceived to be less important. In providing MCID estimates, many respondents stated that they generated these based upon changes in the items they perceived to be most important. Many of these estimates may, therefore, reflect a lower bound of a meaningful difference, but a larger decrease may be necessary if changes are distributed across all DBC-P items.

## Data Availability

The datasets presented in this study can be found in online repositories. The names of the repository/repositories and accession number(s) can be found below: https://osf.io/v6kud/files/osfstorage OSF project: osf.io/v6kud.
