# Supplementary material for: Estimating a minimum clinically important difference for the Developmental Behaviour Checklist – parent report
Source: Front Psychiatry. 2025 Aug 15;16:1612911. doi: 10.3389/fpsyt.2025.1612911 (PMC12395345; doi:10.3389/fpsyt.2025.1612911)
Supplement: Supplementary file 1 [file Supplementaryfile1.docx]

**Supplementary materials A: Developmental Behaviour Checklist vignettes**

**Case 1 – Anna: Behavioural difficulties in a 6-year-old girl**

Anna is a 6-year-old child who lives with her mother, father, and two siblings. She has a history of developmental delays, particularly in spoken language and toilet-training. Her teacher reported that she seems to have some difficulty playing with other children and is often on her own in the classroom. She finds it easier to join in structured activities with small groups of other children during class activities.

Anna was referred for assessment based on behavioural difficulties at home and at school. An assessment at age 5 indicated that she had a mild learning disability. To find out more about her emotional and behavioural difficulties, Anna's mother completed the Developmental Behaviour Checklist.

This showed that Anna’s current Total Behaviour Problem Score on the DBC was 42 out of a maximum score of 192.

Examining the items revealed that many of Anna's difficulties were related to possible anxiety (e.g., Is excessively distressed if separated from familiar person, Cries easily for no reason or over small things, Refuses to go to school, Mood changes).

Below are the scores on the DBC completed by Anna’s mother.

Imagine that Anna is receiving an intervention designed to reduce behavioural and emotional difficulties, particularly the difficulties with interactions with others and anxiety described above.

Look at the individual items on the DBC. For you to feel that the intervention had a meaningful beneficial effect for Anna and her family, what is the smallest possible change in the DBC total score that you think is needed? This would be the smallest number of points in your view that the score would need to decrease by for there to be a meaningful improvement for Anna.

**Case 2 – Ben: Twelve-Year-Old Boy With Mild Learning Disability**

Ben is a boy with a mild learning disability who was referred for assessment by his teacher at a special school for individuals with mild to moderate learning disability. Ben’s mother reported that he has always had difficulty paying attention and focusing on tasks, and she regularly has to remind him to keep focused on a task. However, recently these behaviours have become worse.

At school, Ben gets up from his seat during activities and sometimes leaves the classroom. In addition to struggling to complete set tasks at school, he is also having difficulties with completing daily chores at home. In the morning, he struggles to organise his school things along with other tasks to be ready in time to leave for school. His mother explained that she has to work hard to keep him on task throughout the morning.

Ben's mother was asked to complete the Developmental Behaviour Checklist.

This showed that Ben’s current Total Behaviour Problem Score on the DBC is 55 out of a maximum score of 192.

When reviewing the completed DBC2-P, it became clear that Ben's behaviour was problematic at home and school. Looking closely at the items revealed that many behaviours associated with problems with inattention and overactivity were endorsed with a rating of 2 (Very true or often true). These included: Is easily distracted from tasks; Is irritable; Is impulsive, acts before thinking; Deliberately runs away; and Is very active or restless.

Below are the current DBC scores for Ben. Imagine that Ben is receiving an intervention designed to reduce behavioural and emotional difficulties, particularly the difficulties related to inattention and overactivity described above.

Look at the individual items on the DBC. For you to feel that the intervention had a meaningful beneficial effect for Ben and his family, what is the smallest possible change in the DBC total score that you think is needed? This would be the smallest number of points in your view that the score would need to decrease by for there to be a meaningful improvement for Ben.

**Case 3 – Kathy: Adolescent with Severe Learning Disability**

Kathy is a 16-year-old girl with a severe learning disability who is non-verbal and lives with a foster family. She has lived with this family for the past 18 months. She had experienced several traumatic incidents of abuse by an extended family member in the past and has a diagnosis of posttraumatic stress disorder. Kathy has developed a close attachment to her foster mother and often refuses assistance from other family members.

Recently, Kathy’s foster parents and teachers became concerned that she was showing increased incidents of self-injury (scratching and hitting self, banging her head) resulting in bruising on her legs and torso, and red welts on her lower arms. She had also shown increasingly aggressive (verbal and physical) behaviours towards others at home and school.

The DBC2-P was completed by her foster mother. This showed that Kathy’s current Total Behaviour Problem Score on the DBC is 61 out of a maximum score of 192, suggesting a serious concern about emotional and behavioural difficulties. Of particular note were high scores on items associated with depression, with the following items scored as 2 (Very true or often true): Appears depressed, down, or unhappy; Has a loss of appetite; Sleeps too little, has disrupted sleep; Whines or complains a lot; Is tense, anxious, or worried. Further questioning revealed that the symptoms had begun following the death of her mother, a significant attachment figure who visited her regularly.

Below are the current DBC scores for Kathy. Imagine that Kathy is receiving an intervention designed to reduce behavioural and emotional difficulties, particularly the depression-related difficulties described above.

Look at the individual items on the DBC. For you to feel that the intervention had a meaningful beneficial effect for Kathy and her family, what is the smallest possible change in the DBC total score that you think is needed? This would be the smallest number of points in your view that the score would need to decrease by for there to be a meaningful improvement for Kathy.

**Case 4 - Tim: 11-year-old boy with severe learning disability**

Tim is an 11-year-old boy living with his mother, father, and 8-year-old sister. He has a diagnosis of severe learning disability and is non-verbal. Six months ago Tim moved to a new school.

Tim has always had some repetitive behaviours (e.g., flicked lights on and off, and insisted on objects being placed in specific places) and would become a little agitated if these activities were interrupted. However, over the past six months, the distress he displayed in response to changes in his routines and environment has increased significantly. Tim has begun screaming and hitting his mother, sister, and teacher if they disrupt his routines or ask him to complete tasks. This is particularly problematic in the morning because it he is frequently not ready on time to go to school. The family are concerned that Tim's behaviour might jeopardize his placement at the school.

The DBC2-P was completed by Tim's mother. This showed that Tim’s current Total Behaviour Problem Score on the DBC was 64 out of a maximum score of 192, suggesting a high degree of behavioural and emotional difficulties.

Below are the current DBC scores for Tim. Imagine that Tim is receiving an intervention designed to reduce behavioural and emotional difficulties, particularly those described above,

Look at the individual items on the DBC. For you to feel that the intervention had a meaningful beneficial effect for Tim and his family, what is the smallest possible change in the DBC total score that you think is needed? This would be the smallest number of points in your view that the score would need to decrease by for there to be a meaningful improvement for Tim.

**Case 5 – Adam: 7-year-old boy with mild learning disability**Adam is a 7-year old boy with a mild learning disability. He lives at home with his mother and his 4-year-old sister and attends a mainstream school. He communicates verbally but has difficulty understanding complex information and instructions.

Much of the time, Adam gets on well with other children and adults. He is often affectionate, playful, and friendly. However, recently Adam's mother and teachers have been struggling to manage his behaviour some of the time. 

In particular, when asked to do certain tasks, Adam can become distressed and angry. This includes when he is asked to do learning tasks at school that he is uninterested in and require him to stop a preferred activity, or when he is asked to help with tasks at home such as doing chores. When this happens, Adam will often shout at his mother or teachers and will not follow instructions. More recently, Adam has become more physically aggressive, kicking and pushing his mother, teachers, or other children at school when he is upset. Several times, he has also damaged items including smashing several bowls and a vase during an argument.

Adam's current Total Behaviour Problem Score on the DBC is 40 out of a maximum score of 192.

Below are the current DBC scores for Adam. Imagine that Adam is receiving an intervention designed to reduce behavioural and emotional, particularly the aggressive behaviour described above.

Look at the individual items on the DBC. For you to feel that the intervention had a meaningful beneficial effect for Adam and his family, what is the smallest possible change in the DBC total score that you think is needed? This would be the smallest number of points in your view that the score would need to decrease by for there to be a meaningful improvement for Adam.

**Supplementary materials B: Effect of excluding Wright et al., (2011) (29)**

**ANCOVA meta-analysis of randomised-controlled trials excluding Wright et al. (2011)**

Mean difference = -4.59, standard error= 2.04, 95% CI = -8.58 to –0.59, p= .02, k=14, tau^2^= 37.66, I^2^= 67.01%. Figures 1 and 2 show a forest plot and funnel plot respectively for the ANCOVA meta-analysis RCTs.

**Figure 1**


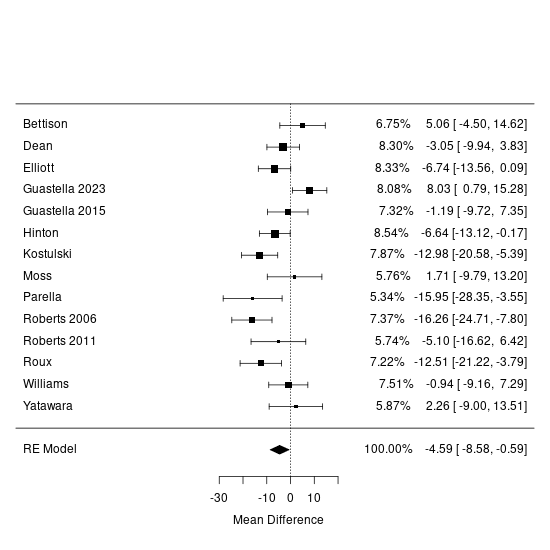
*Forest plot illustrating random effects ANCOVA meta-analysis of RCTs without Wright et al. (2011)*


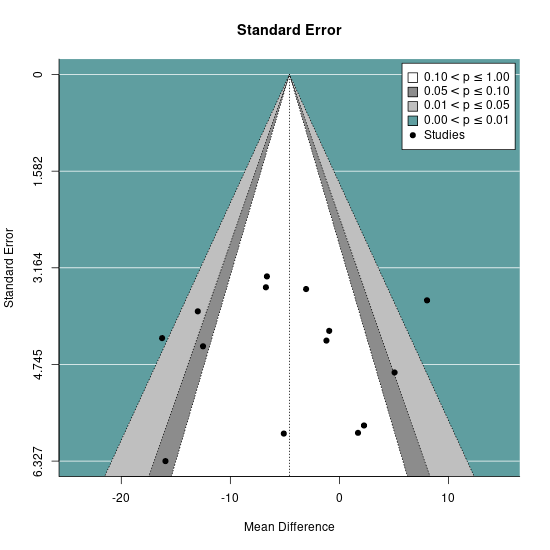
**Figure 2**

*Funnel plot for the random effects ANCOVA meta-analysis of RCTs without Wright et al. (2011)*

**Meta-analysis of randomised-controlled trials final outcome scores excluding Wright et al. (2011)**

Mean difference = -2.82, standard error= 2.19, 95% CI= -7.11 to 1.47, p= .02, k=14, tau^2^= 18.11, I^2^= 27.01%. Figures 3 and 4 show a forest plot and funnel plot respectively for the ANCOVA meta-analysis RCTs.

**Figure 3**


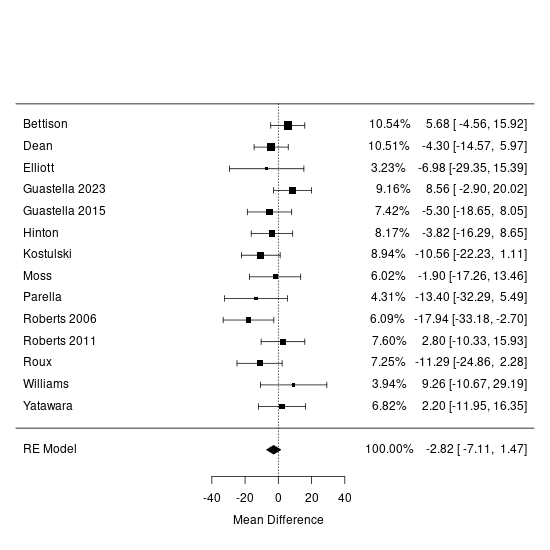
*Forest plot illustrating random effects meta-analysis of final scores in RCTs* *without Wright et al. (2011)*

**Figure 4**


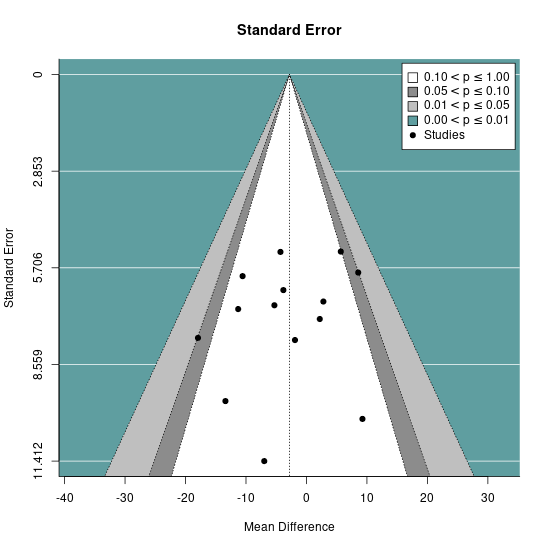
*Funnel plot for the random effects meta-analysis of final scores* *without Wright et al. (2011)*

**Supplementary Materials C**

*Anchor-based MCID estimates by individual case*

| **Case** | **Baseline DBC total score** | **Parents** | | | **Professionals** | | |
| --- | --- | --- | --- | --- | --- | --- | --- |
|  |  | **Mean (SD)** | **Median (IQR)** | **Range** | **Mean (SD)** | **Median (IQR)** | **Range** |
| 1 - Anna | 42 | 4.33(1.97) | 5(2.25) | 1, 6 | 8.89(4.04) | 7(8) | 4, 14 |
| 2- Ben | 55 | 8.00(4.53) | 6(5) | 4, 15 | 9.89(6.66) | 10(10) | 3, 22 |
| 3- Kathy | 61 | 6.80(4.76) | 6(2) | 3, 15 | 12.78(6.78) | 12(10) | 4, 22 |
| 4- Tim | 64 | 8.57(6.40) | 6(6) | 3, 21 | 8.89(4.76) | 8(4) | 3, 19 |
| 5- Adam | 40 | 6.67(4.32) | 5.5(2.5) | 3, 15 | 7.45(4.78) | 6(4) | 3, 17 |
